# The Effect of Gender and Social Capital on the Dual Burden of Malnutrition: A Multilevel Study in Indonesia

**DOI:** 10.1371/journal.pone.0103849

**Published:** 2014-08-25

**Authors:** Masoud Vaezghasemi, Ann Öhman, Malin Eriksson, Mohammad Hakimi, Lars Weinehall, Hari Kusnanto, Nawi Ng

**Affiliations:** 1 Department of Public Health and Clinical Medicine, Division of Epidemiology and Global Health, Umeå University, Umeå, Sweden; 2 Umeå Centre for Global Health Research, Umeå University, Umeå, Sweden; 3 Umeå Centre for Gender Studies, Umeå University, Umeå, Sweden; 4 Centre for Health and Nutrition Research Laboratory, Faculty of Medicine, Gadjah Mada University, Yogyakarta, Indonesia; 5 Centre for Environmental Studies, Faculty of Medicine, Gadjah Mada University, Yogyakarta, Indonesia; Kenya Medical Research Institute - Wellcome Trust Research Programme, Kenya

## Abstract

**Introduction:**

The paradoxical phenomenon of the coexistence of overweight and underweight individuals in the same household, referred to as the “dual burden of malnutrition”, is a growing nutrition dilemma in low- and middle-income countries (LMICs).

**Aims:**

The objectives of this study were (i) to examine the extent of the dual burden of malnutrition across different provinces in Indonesia and (ii) to determine how gender, community social capital, place of residency and other socio-economic factors affect the prevalence of the dual burden of malnutrition.

**Methods:**

The current study utilized data from the fourth wave of the Indonesian Family Life Survey (IFLS) conducted between November 2007 and April 2008. The dataset contains information from 12,048 households and 45,306 individuals of all ages. This study focused on households with individuals over two years old. To account for the multilevel nature of the data, a multilevel multiple logistic regression was conducted.

**Results:**

Approximately one-fifth of all households in Indonesia exhibited the dual burden of malnutrition, which was more prevalent among male-headed households, households with a high Socio-economic status (SES), and households in urban areas. Minimal variation in the dual burden of malnutrition was explained by the community level differences (<4%). Living in households with a higher SES resulted in higher odds of the dual burden of malnutrition but not among female-headed households and communities with the highest social capital.

**Conclusion:**

To improve household health and reduce the inequality across different SES groups, this study emphasizes the inclusion of women's empowerment and community social capital into intervention programs addressing the dual burden of malnutrition.

## Introduction

As the gross national product (GNP) of low- and middle-income countries (LMICs) increases, the overweight burden shifts to the populations with a low socio-economic status (SES), among whom the underweight burden still remains high [1]. The coexistence of both overweight and underweight individuals in the same household, referred to as the “dual burden of malnutrition”, is a growing nutritional dilemma in LMICs. This dilemma is most prevalent in countries experiencing the chronic disease phase of the nutrition transition because of rapid social, economic, and technological changes [2]–[4].

The global burden of overweight mothers and stunted children demonstrates a critical situation in middle-income countries. The highest prevalences of this phenomenon were observed in Guatemala (Latin America), Egypt (Africa), and Uzbekistan (Asia). These results consistently confirm a higher prevalence of a dual burden of malnutrition in rural areas compared with urban areas [5]. Only two studies have observed the dual burden of malnutrition in Indonesia. The first study used data from the 1993 Indonesian Family Life Survey (IFLS) and reported that 11% of households had both overweight and underweight members [2]. The second study analyzed data from the Indonesian Nutrition Surveillance System (NSS) in 2000–2003. This study investigated households with overweight mothers and stunted children and reported a prevalence of 11% in rural areas [6].

The dual burden of malnutrition has been associated with economic factors such as higher SES, household wealth, income, and GDP and socio-demographic characteristics such as family size, age difference between the underweight and overweight members, maternal support, and urban residency [2], [5], [7]–[9]. Despite explicit recognition of the dual burden of malnutrition, knowledge on how gender relations and social capital influence the extent of the dual burden of malnutrition is lacking. Gender is commonly used as a variable for controlling significant differences between men and women in epidemiological studies. However, gender has greater potential to address health issues if this variable is used within a theoretical framework [10]. Gender relations are defined as the relations of power and dominance that structure the life chances of women and men in specific social contexts [11]. Moreover, to complete the theory of justice, Okin argues that equal participation of both men and women in all spheres of human life is remarkably important regardless of whether it is principally the women's or men's domain [12]. Therefore, it is essential to analyze the effect of gender when women take on a typically male role.

In Indonesia, women play a minor role in policy and government decision-making processes [13]. The lack of a female provincial governor and the fact that fewer than 2% of village heads are female indicate that women's representation is even lower at lower levels of government [14]. However, in many parts of Indonesia, women play a critical role in their households, especially in the management of household finances [15]. Although the common perception is that female-headed households are primarily poor and subordinated and encounter numerous social and political discriminations, many of the women generate income through formal employment or informal activities. An active economic role inside and outside the household empowers women to control the finances and authority in the home, which is in contrast with many other predominantly Muslim societies that highly restrict women's economic roles [16]. Therefore, such a culturally, socially, and geographically diverse context provides a unique opportunity to investigate gender and possible inequalities and inequities involved in expanding or diminishing the dual burden of malnutrition in the same households.

Numerous studies have reported a potential link between social capital and health outcomes, including non-communicable diseases (NCDs) [17], [18]. Social capital is generally considered to play an important role in influencing the well being of individuals and households and the development of communities, whether it is an individual attribute or a collective feature. Individual approaches view social capital as something individuals can have more or less access to and describe it as “*the ability of actors to secure benefits by virtues of memberships in networks and other social structures*” [19]. The collective approach views social capital as something characterizing whole communities by levels of trust and civic engagement (e.g., community social capital). This approach defines social capital as “*social networks, the reciprocity that arise from them, and the value of these for achieving mutual goals*” [20]. Collective social capital is often measured as aggregated measures of trust and social participation that does not evidently relate to the living area and therefore the need for a clearer place related measures have been raised [21], [22]. As a country with a long-standing local tradition of community involvement, Indonesia is a unique setting to study social capital. The majority of adult Indonesian women have only elementary education and community participation and social interaction have a huge potential for enriching Indonesian women (as well as men) with additional knowledge that was not received from formal schooling.

## Aims

The objectives of this study were (i) to explore the extent of the dual burden of malnutrition in Indonesia across different provinces and (ii) to determine how gender, community social capital, place of residency and other socio-economic factors affect the dual burden of malnutrition.

## Methods

### Study setting

Indonesia is an archipelago in Southeast Asia with a population of 247 million living in its 34 provinces. Approximately half of the Indonesians live on Java Island. Sixty-nine percent of the population live in rural areas. Muslims form the largest religious group, which accounts for 89% of the population [23].

### Data source

This study utilized the IFLS dataset, which is the only large-scale longitudinal survey available for Indonesia *(* http://www.rand.org/labor/FLS/IFLS.html *)*. The survey was conducted by the RAND Corporation and consists of four waves that were conducted in 1993, 1997, 2000, and 2007–2008. Thirteen out of 27 provinces, which contain 83% of the population, were selected. Enumeration areas (EAs), the primary sampling unit, were randomly selected within each of the provinces. In total, 321 EAs were selected. We used the term “community” to refer to EAs in this study. Households were randomly selected within the EAs. Although the IFLS is a longitudinal survey, weights were constructed both at the individual and household level treating the IFLS as a cross-sectional survey. Therefore, the IFLS was representative of the Indonesian population living in the 13 IFLS provinces in each wave [24]. Only data from the fourth wave (IFLS4) that was collected from 2007–2008 were used in this study. This dataset contains information from 12,048 households and 45,306 individuals of all ages who lived in 13 of the 27 provinces in Indonesia.

### Outcome variables

#### (i) Assessment of overweight and underweight at the individual level

**Figure 1:**
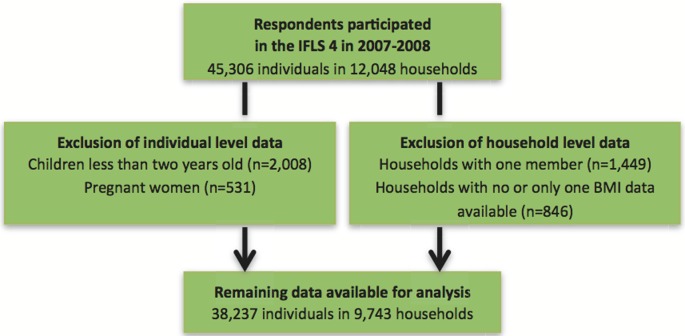
IFLS 4 participants and the exclusion criteria in this study.

Children less than two years old and pregnant women were excluded. As a result, 12,037 households and 42,749 individuals remained in the study (Figure 1). Two trained nurses recorded physical health measurements of the household members, including their height and weight [24]. For adults, we calculated the body mass index (BMI) and used the cut-offs of <18.5 and ≥25 kg/m^2^ to categorize individuals as underweight, normal weight, and overweight/obese [25]. We calculated BMI based on the definition of World Health Organization (WHO) to facilitate the comparison of the findings with other studies conduced in different Asian and African countries. It has been shown that Asian population such as Indonesians (Malays and Chinese ancestry), Singaporean Chinese, Malays and Indians, and Hong Kong Chinese have a higher body fat percent at a lower BMI compared to Caucasians that might lead to higher risk of chronic diseases [26]. For children and adolescents aged 2–18 years old, age- and sex-specific cut-off points established by Cole et al. [27], [28] that correspond to an adult BMI of 18.5 and 25 at the age of 18 were used to determine underweight and overweight, respectively. In these studies the same method (LMS) were used to define overweight and obesity as well as thinness or underweight using data from nationally representative surveys of children in six high and middle-income countries. The LMS method summarizes the distribution of BMI by age and sex in terms of three curves called L (lambda), which expresses the Skewness of BMI distribution, M (mu), which is median BMI by age, and S (sigma), which is the coefficient of variation of BMI. Accordingly, any required BMI centile curve is defined as follows: **M(1+L×S×z)^1/L^**. Where z is the z score corresponding to the required centile and the values of L, M, and S vary with age and sex. The reverse process, of converting a child's BMI to a z score, involves the equation: **z = ((BMI/M)^L^−1)/(L×S)**. The values of L, M, and S are for the child's age and sex.

#### (ii) Assessment of the dual burden of malnutrition at the household level

A household with a dual burden of malnutrition was identified if there was at least one underweight and one overweight member in the household. Households with only one member and households without BMI data or only one available BMI measurement were excluded. Finally, 9,743 households consisting of 38,237 individuals were included in the analysis (Figure 1).

Households were classified into four distinct groups, including (i) “underweight households” with at least one underweight member and no overweight member (28%), (ii) “normal households” without underweight or overweight members (21%), (iii) “overweight households” with at least one overweight and no underweight member (32%), and (iv) “dual burden households” with at least one underweight and one overweight member (19%). In this study, we focus on comparing the “dual burden households” with the other types of households combined.

### Household level variables

#### (i) Male- and female-headed households

Households were classified as male- and female-headed households based on the sex of the household's head reported in the survey. Statistics Indonesia (BPS) defined a head of the household as (i) the person who is actually responsible for the daily needs of a household or (ii) the person who is considered the head of the household [29].

#### (ii) Education level of the household heads

The education level of the household head is based on their highest education level and was categorized into “no schooling”, “elementary”, “secondary”, and “university/college”.

#### (iii) Socio-economic status of households (SES)

We used asset and housing variables to create SES quintiles (1 = poorest and 5 = richest) using a Principal Component Analysis (PCA) [30]. The variables included households' ownership of durable assets (e.g. TV, refrigerator, jewelry, vehicles, and savings certificates or disposal stocks) and infrastructure and housing characteristics (e.g. source of water, drainage, toilet, cooking, type of walls, roof, and floor).

#### (iv)Under 15- and over 60-year-olds

We controlled for the influence of age in the analysis by accounting for the proportion of household members less than 15 years old and greater than 60 years old.

### Community level variables

#### (i) Community social capital

In this study, the collective feature of social capital was measured based on the existence of programs and activities in the community, such as village cooperatives, neighborhood watch programs, village improvements, child development, teen development, elderly programs, youth and family groups, village loans, and health funds in the communities. The responses to each of these indicators were used and summarized with a PCA and the resulting index was divided into tertiles, including lowest, middle, and highest community social capital.

#### (ii) Place of residency

In addition, households were categorized based on living in urban or rural areas to control for the effect of residency on the analysis.

### Statistical analysis

To account for the multistage/cluster sampling design, we used the individual and household weights in all the analyses of individual and household level data, respectively. This was performed using the svyset and svy commands in Stata Version 12.1 (StataCorp, 4905 Lakeway Drive, College Station, Texas 77845, USA). The individual level data were used to calculate the prevalence of underweight and overweight individuals. The data were further collapsed into household level data for subsequent analyses on the dual burden of malnutrition. The results of the descriptive analysis were presented as numbers and weighted percentages with their 95% confidence interval (CI). Prevalences were presented for each province and provinces were ranked based on their 2008 Human Development Index (HDI) [29]. Simple and multiple logistic regression analyses were used to evaluate associations between household and community level factors with the dual burden of malnutrition. The results were expressed as odds ratios (ORs) with a 95% CI. Variables were considered significant at the 0.05 level. To account for the multilevel nature of the data (households within communities), we conducted a multilevel multiple logistic regression to assess the interclass correlation and variations of the outcome variables at a higher level [31]. We estimated a random intercept model to investigate the extent that the dual burden of malnutrition could be explained by community level determinants. The data were stratified by household type (female- or male-headed), community level of social capital (lowest or highest), and place of residency (urban or rural).

## Results

A total of 45,306 individuals within 12,048 household participated in the IFLS4 with a female/male ratio of 0.96. In this study, complete data were obtained from 38,237 individuals within 9,743 households.

### Prevalence of underweight and overweight individuals

The prevalence of underweight and overweight were significantly different among provinces (p<0.001). Underweight was more common in rural areas (20% in rural vs. 18% in urban, p<0.001) and in provinces with a lower HDI. In contrast, overweight was more prevalent in provinces with a higher HDI and in urban areas (25% in urban vs. 17% in rural, p<0.001) (Table 1). Additionally, we determined that underweight was more prevalent among children and overweight/obesity was more prevalent among women (Appendix 1).

**Table 1:** Prevalence (and 95% confidence interval/CI) of underweight and overweight at the individual level in the 13 provinces of Indonesia in 2007.

Provinces (ranked based on their human development index)	Sample size (n = 42755)		Underweight % (95% CI)		Overweight % (95% CI)	
	Rural (n)	Urban (n)	Rural	Urban	Rural	Urban
Jakarta	NA	3199	NA	17 (15–19)	NA	30 (27–32)
Yogyakarta	526	1719	23 (20–27)	18 (15–21)	15 (12–16)	24 (20–28)
North Sumatra	1534	1629	12 (10–15)	15 (12–18)	19 (13–25)	26 (22–29)
West Sumatra	1211	965	20 (16–24)	15(12–18)	20 (14–25)	27 (22–31)
South Sumatra	1449	723	22 (18–26)	20 (17–23)	14 (10–17)	30 (27–33)
Central Java	3052	2328	17 (14–19)	18 (14–21)	18 (16–20)	24 (20–27)
West Java	2415	4172	22 (18–26)	17(15–19)	17 (14–20)	24 (22–26)
Bali	939	1281	15 (11–19)	13 (11–15)	23 (17–30)	27 (22–32)
East Java	3335	2969	23 (19–26)	18 (16–20)	18 (15–21)	25 (22–27)
Lampung	1302	598	20 (18–23)	23 (20–26)	16 (13–19)	17 (12–22)
South Sulawesi	1323	1180	21 (19–24)	23 (18–29)	16 (11–22)	19 (15–23)
South Kalimantan	1199	878	24 (20–28)	22 (17–27)	16 (14–19)	27 (22–31)
West Nusa Tenggara	1704	1125	29 (22–36)	27 (23–30)	9 (7–12)	19 (15–23)
**Total**	**19989**	**22766**	**20 (19–22)** *	**18 (17–19)** *	**17 (16–18)**	**25 (24–26)** *

Note: NA = There is no rural area in the province of Jakarta. The 1990 Census declared this city as a fully urbanized area. To adapt to the growth of Jakarta, all non-urban areas were converted to urban areas.

Provinces were ranked by the human development index from 77.03 in Jakarta to 64.12 in West Nusa Tenggara (35).

*p-value <0.05 (difference among provinces).

### Prevalence of the dual burden of malnutrition among households

**Figure 2:**
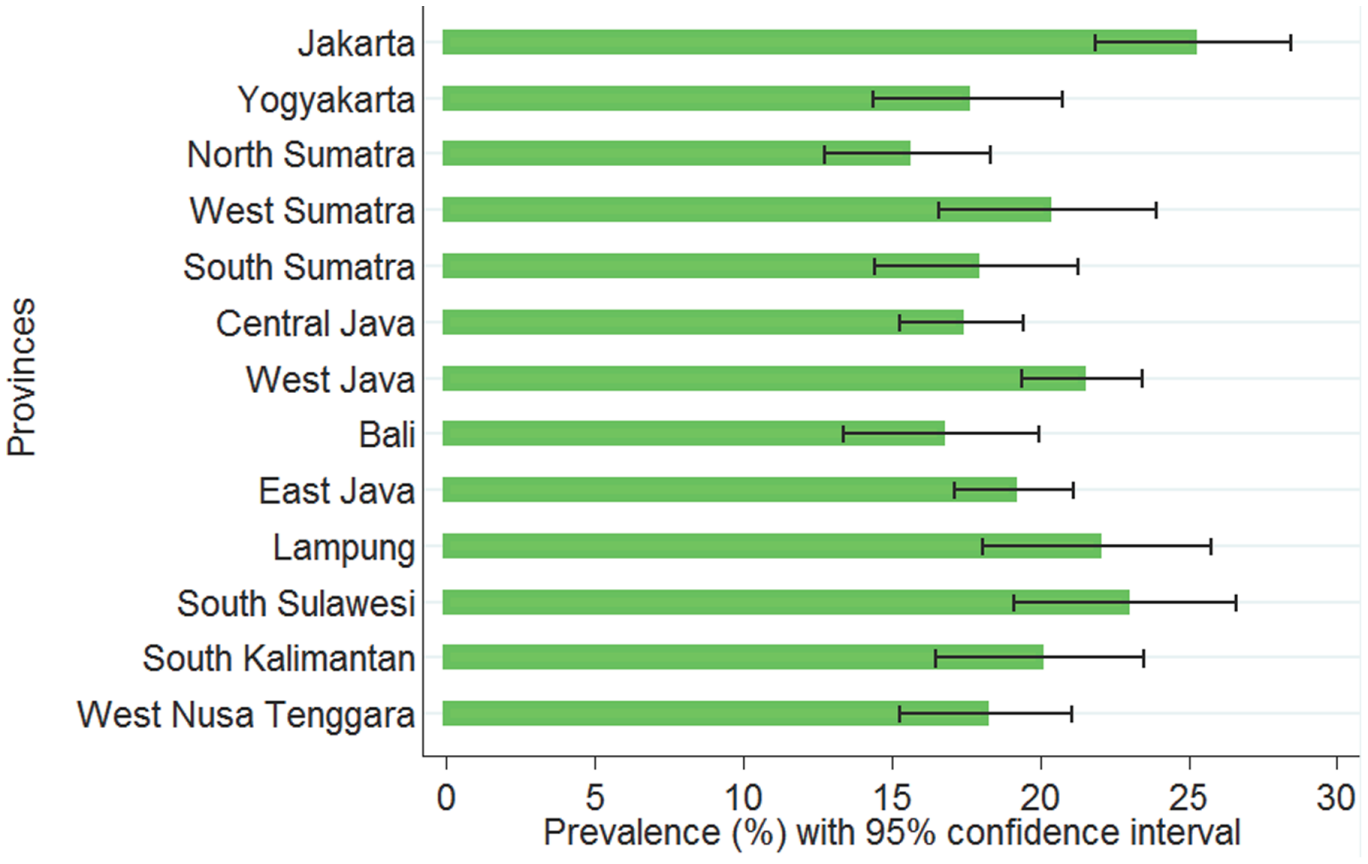
Prevalence of dual burden of malnutrition by province, sorted based on their Human Development Index (Jakarta and West Nusa Tenggara were the provinces with the highest and lowest HDI, respectively.

The percentage of households with a dual burden of malnutrition in Indonesia was 19% and significantly different among provinces (p<0.01). Jakarta (25%), South Sulawesi (23%), and Lampung (22%) had the highest prevalences. North Sumatra (15%), Central Java (17%), and Yogyakarta (17%) had the three lowest percentages of households with a dual burden of malnutrition (Figure 2).

**Figure 3:**
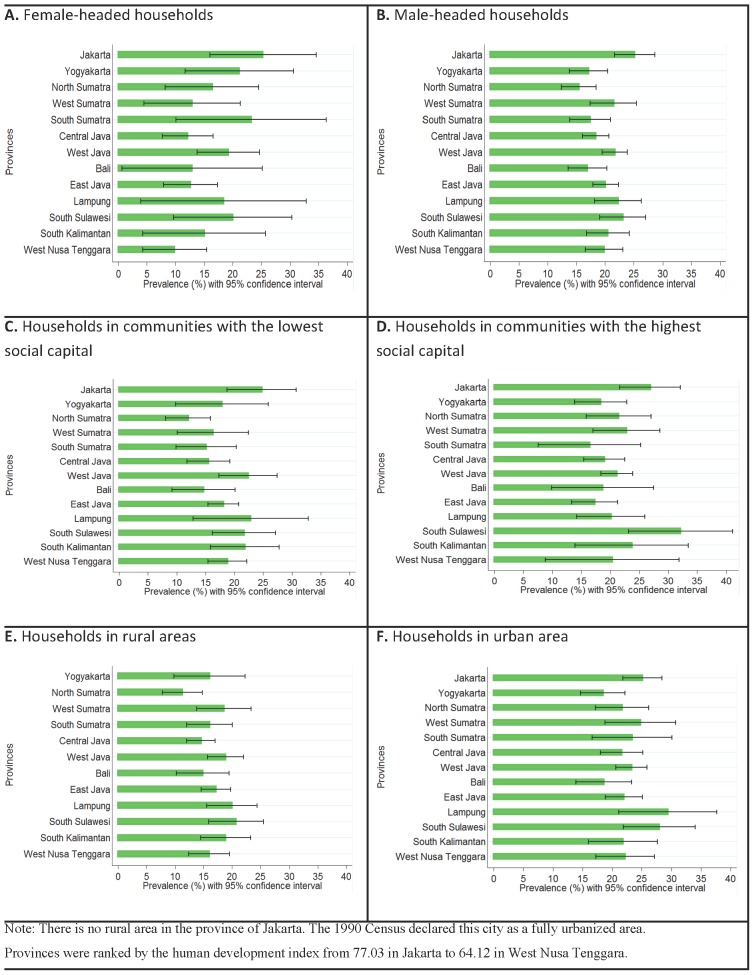
The prevalence of dual burden of malnutrition across provinces in Indonesia in households with different characteristics based on the head of household gender, community social capital, and type of area.

The differences in the prevalence of the dual burden of malnutrition across provinces were more prominent among female-headed households compared with male-headed households (Figure 3A and 3B) and in urban compared to rural areas (Figure 3E and 3F). No significant difference was observed across provinces when we compared households living in communities with the lowest and highest social capital (Figure 3C and 3D). In Jakarta, no differences in the prevalence of the dual burden of malnutrition across these population subgroups were observed, which is in contrast with Central Java, where large differences were observed.

### Determinants of the dual burden of malnutrition

The univariate logistic regression revealed that the dual burden of malnutrition was more prevalent (i) among male-headed households than female-headed households, with prevalences of 20% and 16%, respectively, (ii) among households for which head had a higher education level, and (iii) among households with a high socio-economic status. There was no significant difference between households with less than 50% and more than 50% less than 15- and greater than 60-year-old members. When we analyzed community level characteristics, the prevalence was higher in urban areas compared with rural areas, 23% and 17%, respectively. Although the prevalence was slightly higher in communities with the highest social capital compared with communities with lowest social capital, 20% and 18%, respectively, the difference was not statistically significant (Table 2).

**Table 2:** Household and community level characteristics associated with the dual burden of malnutrition in Indonesia in 2007.

Household characteristics	Total number of households with dual burden n (%)	Simple single level logistic regression	Multilevel multiple logistic regression
**Overall (n = 9743 households)**	1940 (19)		
***Fixed-effects model*** ** (OR with 95% CI)**			
*Household level variables*			
**Household head**			
Male (n = 8498)	1739 (20)	1	1
Female (n = 1232)	199 (16) *	0.74 (0.62–0.88)	0.74 (0.62–0.88)
**Household head education**			
No schooling (n = 693)	114 (15)	1	1
Elementary school (n = 3905)	750 (19)	1.29 (0.94–1.78)	1.06 (0.85–1.33)
Secondary school (n = 3520)	737 (20)	1.40 (1.01–1.93)	0.99 (0.78–1.25)
University/college (n = 851)	204 (24) *	1.74 (1.21–2.51)	1.07 (0.80–1.42)
**Household SES**			
1 poorest (n = 1969)	275 (13)	1	1
2 (n = 1932)	353 (18)	1.40 (1.14–1.72)	1.29 (1.08–1.56)
3 (n = 2011)	435 (21)	1.76 (1.44–2.16)	1.61 (1.34–1.93)
4 (n = 1897)	438 (22)	1.85 (1.49–2.30)	1.65 (1.36–1.99)
5 richest (n = 1930)	437 (23) *	1.95 (1.57–2.41)	1.61 (1.31–1.99)
**Less than 15- and greater than 60-year-olds**			
< 50% (n = 5787)	1115 (19)	1	1
≥50% (n = 3956)	825 (20)	1.09 (0.98–1.22)	1.16 (1.04–1.29)
*Community level variables*			
**Community social capital**			
1 Lowest (3490)	651 (18)	1	1
2 Middle (3042)	609 (20)	1.13 (0.94–1.36)	1.05 (0.90–1.22
3 Highest (3211)	680 (20)	1.16 (0.98–1.38)	1.02 (0.88–1.18)
**Residency**			
Urban (n = 5002)	1139 (23)	1	1
Rural (n = 4741)	801 (17) *	0.69 (0.60–0.78)	0.77 (0.68–0.88)
***Random effects***			
Null model community random variance (SE)			0.092
Community random variance (SE)	**--------**	**--------**	0.061
Variance Partition Coefficient (%)	**--------**	**--------**	2%
Proportional changes in the variance (PCV)	**--------**	**--------**	34%
Median Odds Ratio (MOR)	**--------**	**--------**	1.26
Interval Odds Ratio (IOR) for residency	**--------**	**--------**	0.49–1.20
Sorting out index for residency	**--------**	**--------**	22%

*p-value<0.05.

Variables were considered significant at the 0.05 level.

The multilevel multiple logistic regression (only fixed-effect) analysis revealed that being in a female-headed household and living in a rural area was significantly associated and had a protective effect. The odds of having a dual burden of malnutrition were higher in households with a higher SES. Community social capital was not significantly associated with the dual burden of malnutrition in any of the regression analyses (Table 2).

The results from the random-effect portion of the multilevel multiple logistic regression models indicated that (i) a small amount of the variation in the prevalence of the dual burden of malnutrition (VPC 2%) was explained by the community level differences and the place of residency, (ii) the proportional change in variance (PCV) was 34%, (iii) the median odds ratio (MOI) was 1.26, (iv) the interval odds ratio (IOR) was 0.49–1.20, and (v) the sorting out index was 22%. Additionally the differences in province level were controlled for in this model, but no significant change was observed.

After stratifying the multilevel multiple logistic regression analyses by household head, community social capital, and place of residency, the results from the fixed-effect portion of Table 3 illustrate that (i) being in a female-headed household exhibited a protective effect regardless of community social capital and place of residency, (ii) being in a household in the highest SES quintiles was not significantly associated with the dual burden of malnutrition in female-headed households and households having the highest community social capital, (iii) among households in communities with the lowest social capital, being in the highest SES quintiles increased the odds of a household having a dual burden of malnutrition (2.07, 1.45–2.96) compared with those in the lowest SES quintiles, (iv) living in households with the highest SES quintiles also increased the odds of having a dual burden of malnutrition by 54% in urban areas and by 67% in rural areas compared with those in the lowest SES quintiles.

**Table 3:** Household and community level characteristics associated with dual burden of malnutrition in Indonesia in 2007 across households with different characteristics.

	Model stratified by household head		Model stratified by community social capital		Model stratified by place of residency	
	Female	Male	Lowest	Highest	Rural	Urban
**Fixed-effects (OR with 95% CI)**						
*Household level variables*						
**Household head**						
Male (n = 8498)	--------	--------	1	1	1	1
Female (n = 2332)	--------	--------	0.68 (0.51–0.92)	0.76 (0.57–1.00)	0.74 (0.57–0.96)	0.74 (0.59–0.93)
**Household head education**						
No schooling (n = 693)	1	1	1	1	1	1
Elementary school (n = 3905)	1.04 (0.67–1.60)	1.06 (0.81–1.38)	1.22 (0.85–1.74)	0.84 (0.56–1.27)	1.00 (0.75–1.34)	1.12 (0.78–1.61)
Secondary school (n = 3520)	1.03 (0.63–1.70)	0.99 (0.75–1.30)	1.06 (0.73–1.55)	0.83 (0.55–1.27)	0.94 (0.69–1.29)	1.04 (0.72–1.49)
University/college (n = 851)	1.27 (0.58–2.79)	1.04 (0.76–1.44)	0.95 (0.58–1.55)	1.02 (0.64–1.64)	1.15 (0.73–1.80)	1.07 (0.71–1.60)
**Household SES**						
1 poorest (n = 1969)	1	1	1	1	1	1
2 (n = 1932)	0.93 (0.54–1.60)	1.36 (1.12–1.66)	1.26 (0.93–1.69)	1.26 (0.90–1.77)	1.28 (1.02–1.60)	1.26 (0.91–1.75)
3 (n = 2011)	1.09 (0.65–1.82)	1.70 (1.40–2.07)	1.78 (1.32–2.40)	1.29 (0.92–1.79)	1.57 (1.24–1.99)	1.56 (1.14–2.13)
4 (n = 1897)	1.48 (0.88–2.50)	1.68 (1.37–2.06)	1.85 (1.35–2.54)	1.36 (0.97–1.91)	1.70 (1.30–2.22)	1.54 (1.13–2.10)
5 richest (n = 1930)	1.28 (0.71–2.33)	1.68 (1.34–2.09)	2.07 (1.45–2.96)	1.20 (0.84–1.72)	1.67 (1.18–2.37)	1.54 (1.12–2.10)
**Less than 15- and greater than 60-year-olds**						
<50% (n = 5787)	1	1	1	1	1	1
≥50% (n = 3956)	0.68 (0.50–0.94)	1.25 (1.11–1.40)	1.25 (1.04–1.51)	1.07 (0.90–1.29)	1.15 (0.98–1.36)	1.17 (1.01–1.34)
*Community level variables*						
**Community Social Capital**						
1 Lowest (3490)	1	1	--------	--------	1	1
2 Middle (3042)	1.21 (0.82–1.79)	1.03 (0.88–1.21)	--------	--------	1.07 (0.85–1.34)	1.00 (0.83–1.20)
3 Highest (3211)	1.12 (0.76–1.66)	1.00 (0.86–1.17)	--------	--------	1.10 (0.87–1.39)	0.94 (0.79–1.12)
**Residency**						
Urban (n = 5002)	1	1	1	1	--------	--------
Rural (n = 4741)	0.82 (0.57–1.17)	0.77 (0.67–0.88)	0.74 (0.59–0.94)	0.79 (0.65–0.98)	--------	--------
***Random effects***						
Null model community random variance (SE)	1.36	0.096	0.177	0.005	0.094	0.016
Community random variance (SE)	1.51	0.065	0.137	4.030	0.090	0.011
Variance Partition Coefficient (%)	0%	2%	4%	0%	3%	0%
PCV	−11%	32%	22%	-------1	4%	31%
MOR	1.00	1.27	1.42	1.00	1.33	1.10
IOR (residency)	0.09–7.58	0.48–1.22	0.38–1.45	0.071–8.84	--------	--------
Sorting out index (residency)	45%	23%	28%	42%	--------	--------

1 Not Available.

Variables were considered significant at the 0.05 level.

The random-effect portion of Table 3 indicates that less than 4% of the variation in the prevalence of the dual burden of malnutrition is explained by community level differences in the regression analyses for all the sub-groups.

## Discussion

The main findings of this study include the following: (i) approximately one-fifth of all households in Indonesia exhibit a dual burden of malnutrition with significant differences in the prevalence across provinces; (ii) only a small amount of the variation in the dual burden of malnutrition could be explained by the community level differences (<4%), while the remaining 96% could be explained by differences between households; and (iii) the main determinants of the dual burden of malnutrition are having a male head of the household, living in households with a higher SES, and living in an urban area. Moreover, the level of community social capital is not significantly associated with the prevalence of the dual burden of malnutrition, and households with higher SES levels are positively associated with a dual burden of malnutrition regardless of whether the household is located in an urban or a rural area. However, these associations are only significant in male-headed households and households in communities with the lowest social capital.

The prevalence of a dual burden of malnutrition has nearly doubled during the last fifteen years according to data from another conducted study in Indonesia using the 1993 IFLS data, which had reported a prevalence of 11% [2]. Moreover, studies in other LMICs have revealed that this phenomenon is not unique for Indonesia. In 2006, the Food and Agriculture Organization of the United Nations (FAO) documented the dual burden of malnutrition in six LMICs, including China, Egypt, India, Mexico, the Philippines, and South Africa. India and the Philippines exhibited the highest prevalence of underweight, while over nutrition is an emerging problem, especially in urban areas. In the Philippines, 27% of children under five years of age are underweight, while 27% of women are overweight or obese [32]. In this study, interventions aimed at the dual burden should be prioritized in India and the Philippines, while overweight and obesity should be targeted in Egypt, Mexico, and, to a lesser degree, China and South Africa. Because India and the Philippines have the lowest GDPs among these countries, comparing the GDP may reveal that when the economy develops, the problem of dual burden of malnutrition arises in countries with low GDP, while it disappears in countries with a higher GDP as the transition moves toward overnutrition and an obesity epidemic [8].

In agreement with our findings, several other studies have reported the association between a higher SES, income or GDP and urban residency with a dual burden of malnutrition in LMICs [2], [5], [7], [8]. In LMICs, as the country's GDP increases, overweight emerges while underweight remains a prominent health problem resulting in the dual burden of malnutrition [1]. Therefore, at a macro level, the population's diet structure shifts combined with decreased physical activity and increased sedentary jobs, the introduction of labor-saving technology as well as urbanization deteriorates the current situation [3], [33]. At the micro level, as the economic development proceeds, households' per capita income and expenditures increase. Consequently, an increase in total calorie intake and higher consumption of carbohydrates, proteins, and fats results in unbalanced dietary habits [34]. However, improvement in the spouse's education has a positive and significant influence on the consumption of some nutrients, including protein, vitamins, and the substitution of fat for carbohydrates [34].

To our knowledge, no study has investigated the societal aspect of dual burden of malnutrition such as female-headed households and community social capital as well as applying related theories in explaining the statistical significances. In this study, we identify the inequality in the distribution of double burden of malnutrition across different provinces in Indonesia, and the prevalence ranges from 15% in North Sumatera province to 25% in Jakarta province. We also use multilevel approach and identify that only 4% of the variation in dual burden of malnutrition could be attributed to the unobserved factors at community level. These are novice knowledge that has not been addressed in previous research. A recently published article, however, examined the association between intra-household inequalities and dual burden of malnutrition in Indonesia using longitudinal IFLS data [35]. This study reported lower nutritional inequality among households with female-headed households and suggested follow-up research on more detailed data in order to understand the gender role in male-headed households. Another study in the Andhra Pradesh State of India reported a protective effect of maternal social capital and dual burden of malnutrition [9]. This study argues that a larger number of people available to supports mothers results in an increased possibility of information sharing and financial, maternal, and psychological support. These events lead to better childcare and have a protective effect against underweight child and overweight mother households. Moreover, the results from a case study by Quisumbing and Maluccio in Bangladesh, Indonesia, Ethiopia, and South Africa revealed a positive and significant effect on expenditure allocation to the next generation, including education and children's clothing, when the households' assets are controlled by women [36]. While the authors did not investigate the nutritional status of children, they determined that children are better off in households where women have bargaining power and decision-making power.

Several studies emphasize the positive influence on children's health and condition when women are empowered in the households. Additionally, underweight children contribute significantly to the dual burden phenomenon; thus, if empowerment of women improves children's health, this empowerment will also, in turn, protect households against the dual burden of malnutrition. This hypothesis may explain the protective effect of the female-headed households on the dual burden of malnutrition reported in the present study even after controlling for the head's education and household SES. Taken together, it is “gendered power relations” within the households that affect household health when women take the position that is typically a male role [11], [37]. This theoretical model might be useful in describing and analyzing how power structure functions in a specific context, as well as in exploring how gender theory may explain the intricate relationship between gender and health. However, the mechanisms of how “gendered power relations” affect the dual burden of malnutrition have not been elucidated further.

Additionally, this study presents the effects of different household SES on the dual burden of malnutrition at the household level compared with the household head gender and the level of community social capital where the households were located. Household SES does not significantly affect the dual burden of malnutrition in households with a female head and situated in communities with a high social capital. These results suggest that empowering women and improving community social capital could reduce inequalities across different SES groups. These findings are supported by studies in Indonesia [15], [38] and in other developing countries [39], [40]. Putnam emphasized the relationship between social capital and economic development [41]. He argues that the dense social networks in the rapidly growing economies of East Asia are based on the extended family or close-knit ethnic communities that foster trust, lower transaction costs, and increased information and innovation, which can also be used as a form of financial capital. However, in the present study, understanding how community social capital functions in reducing the harmful effects of high SES on the dual burden of malnutrition requires further examination.

The current study has several strengths, including a large sample size, a low percentage of missing data and a high response rate, which provide sufficient statistical power to make robust inferences of the extent of the dual burden of malnutrition in Indonesia. Therefore, the probability of bias in estimating the odds ratios is reduced. Conducting a multilevel analysis at the country level on a novel research topic, such as the dual burden of malnutrition, is an additional strength of the present study. Considering the role of gender and community social capital with other household and community level factors provides an opportunity to examine the dual burden of malnutrition from different societal angles and to identify niches for potential interventions from a social perspective.

Some limitations of this study need to be considered when interpreting the results. Drawing conclusions about causality was not possible because of the cross-sectional study design. Dual burden of malnutrition was not separately compared with other types of households such as overweight, underweight, and normal weight. Even though, such comparisons were not within the scope of the current study, further detailed analyses could better help understanding the determinants of the dual burden of malnutrition. For instance, in the study by Roemling and Qaim, fewer significant effects were observed when they compared dual burden households with overweight households, separately. This indicates that dual burden households are more similar to overweight households compared to normal weight households [35]. In addition, the reported relationships may arise from the influence of unobserved household and community level factors associated with both the dependent and independent variables. Factors such as household diets and nutritional intake as well as community level strategies and measurements to prevent malnutrition were not available in this study. Children less than two years old were excluded because information on age based on month was not available. Therefore, it was not possible to assess their nutritional status. As children less than two years old generally constitute the majority of underweight members in households, exclusion of this group might influence the estimates reported in this study.

## Conclusion

The dual burden of malnutrition in Indonesia doubled between 1993 and 2008 and is unequally distributed. Inequalities in the dual burden of malnutrition in Indonesia exist across populations with different economic developments. However, these gaps are decreased in populations with access to community social capital. Furthermore, the sex of household's head as a proxy for gender relations plays a significant role in influencing the existing problem at the household level. Therefore, to address the dual burden of malnutrition in Indonesia, this study emphasizes the importance of women's empowerment and improvement of community social capital in community intervention programs.
